# A bibliometric analysis of the global research on biosimilars

**DOI:** 10.1186/s40545-018-0133-2

**Published:** 2018-03-27

**Authors:** Akram Hernández-Vásquez, Christoper A. Alarcon-Ruiz, Guido Bendezu-Quispe, Daniel Comandé, Diego Rosselli

**Affiliations:** 1 Universidad Privada del Norte, Lima, Peru; 2 Facultad de Medicina, Universidad Ricardo Palma, Lima, Peru; 3 Universidad Peruana Cayetano Heredia, Lima, Peru; 4 Institute for Clinical Effectiveness and Health Policy (IECS), Buenos Aires, Argentina; 5 Departamento de Epidemiología Clínica y Bioestadística, Facultad de Medicina, Pontificia Universidad Javeriana, Bogotá, Colombia

**Keywords:** Biosimilar pharmaceuticals, Bibliometrics, Biomedical research (source: MeSH NLM)

## Abstract

**Background:**

Biosimilars could be a promising option to help decrease healthcare costs and expand access to treatment. There is no previous evidence of a global bibliometric analysis on biosimilars. Therefore, we aimed to assess the quantity and quality of worldwide biosimilars research.

**Methods:**

We performed a bibliometric analysis using documents about biosimilars published until December 2016 in journals indexed in Scopus. We extracted the annual research, languages, countries, journals, authors, institutions, citation frequency, and the metrics of journals. The data were quantitatively and qualitatively analyzed using Microsoft Excel 2013. Additional information about authors' participation was obtained using the R-package Bibliometrix. Publication activity was adjusted for the countries by population size. Also, author co-citation analysis and a term co-occurrence analysis with the terms included in the title and abstract of publications was presented as network visualization maps using VOSviewer.

**Results:**

A total of 2330 biosimilar-related documents identified in the Scopus database, most of them were articles (1452; 62.32%). The number of documents published had an exponential increased between 2004 and 2016 (*p* < 0.001). The United States was the country with the highest production with 685 (29.40%) documents followed by Germany and UK with 293 (12.58%) and 248 (10.64%), respectively. Switzerland (11.05), Netherlands (5.85) and UK (3.83) showed the highest per capita ratio. The highest citation/article ratio were for the Netherlands (28.06), Spain (24.23), and France (20.11). *Gabi Journal* published 73 (3.13%) documents; both *Biopharm International* and *Pharmaceutical Technology* and *Mabs,* 41 (1.76%). Three out of top ten journals were Trade publications. Amgen Incorporated from the USA was the most prolific institution with 51 documents followed by Pfizer Inc. with 48. Terms about specific diseases and drugs were found in recent years, compared with terms such as legislation, structure, protein, dose and generic in the early years.

**Conclusions:**

Research production and publication of documents on biosimilars are increasing. The majority of publications came from high-income countries. The trends in terminology use are according to state of the art in the topic, and reflects the interest in the utilization of biosimilars in diseases who are expected to obtain benefits of its use.

**Electronic supplementary material:**

The online version of this article (10.1186/s40545-018-0133-2) contains supplementary material, which is available to authorized users.

## Background

A biosimilar, or “similar biological product”, is a highly similar product to an already approved biological product regarding structure, function, potency, quality, clinical efficacy and safety [1]. Currently, there are already 5 and 39 biosimilars products approved in the United States [2] and Europe [3], respectively. The number of approved biosimilars might increase in the next year due to manifest interest from different healthcare systems and international organizations [4].

The development of biological products represented a significant advance in the therapy of many diseases that did not have effective treatments. Nonetheless, these products require a vast expenditure of money and resources to develop, leading to an increase in the cost of these therapies for both patients and the health sector [5, 6]. The field of biosimilars could be a promising option to help decrease healthcare costs and expand access to treatment [7] particularly when many patents have already expired or will do so soon [8].

Few studies had evaluated the clinical evidence in support of different biosimilars: there are only three cancer related-biosimilars products whose safety/efficacy have been published [9], while five biosimilars for chronic inflammatory diseases have six clinical trials comparing them with their reference drug [10]. This situation of scarce evidence may lead physicians [11], pharmacists [12] and patients to have low confidence their safety and efficacy [13].

Bibliometric analysis is a useful method to objectively measure current research of a certain subject and its international scientific influence as an aspect of scientific quality [14]. However, to our knowledge, there is no previous evidence of a global bibliometric analysis on biosimilars. Our study aimed to assess the quantity and quality of worldwide biosimilars research.

## Methods

### Study design

We performed a bibliometric analysis using documents published until December 2016 in journals indexed in Scopus (https://www.scopus.com/). While there are a variety of document types, only articles, reviews, editorials and letters were included.

### Source of information

Scopus (Elsevier BV Company, USA) is the largest abstract and citation database of scientific peer-review literature including more than 22,000 titles from international publishers. We decided to use this database because it includes all MEDLINE documents and includes further characteristics such as country of all the authors and citations per document, information that is relevant for this study [15–17].

### Search strategy

A literature search was conducted by a research librarian in Scopus for publications on a single day, October 18, 2017, and used the following MeSH and free terms in the title and abstract field: biosimilar pharmaceutical OR biosimilar*. The validity of the search strategy was tested by manually reviewing retrieved articles.

### Data analysis

All data were collected by two authors and downloaded in csv format (Additional file 1: Dataset). The data were imported to Microsoft Excel 2013 and quantitatively and qualitatively analyzed. The Scopus database presents some disadvantages for bibliometric applications [17]. For this reason, it was necessary to standardize the data. We detected documents mistakenly attributed to the domain of author name and affiliation. Therefore, a standardization was carried out manually by the authors.

Bibliometric indicators were extracted from the data and with the option “Analyze Results” in Scopus, including annual research, languages, countries, journals, authors, institutions, and citation frequency. Scopus Journal Metrics was used to extract the metrics of journals. The contributions of countries were evaluated based on paper and citation numbers, and the research output of each country was adjusted according to population size (https://www.cia.gov/library/publications/the-world-factbook/geos/ag.html). To describe more information about author participation in research production about biosimilars, we use the open-source Bibliometrix R-package (http://www.bibliometrix.org/) to obtain the mean of articles per author, the mean of authors per article, the mean of articles’ citation and the number of articles with only one or more than one author.

Author co-citation analysis (ACA) to analyze the relations among highly cited references and productive authors, and a term co-occurrence analysis with the terms included in the title and abstract of publications was presented as network visualization maps using VOSviewer version 1.6.6 (Leiden University, Leiden, Netherlands) techniques [18].

### Research ethics

The data were downloaded from Scopus and as secondary data, did not involve any interactions with human subjects. There were no ethical questions about the data. Approval of an ethics committee was not necessary.

## Results

**Fig. 1:**
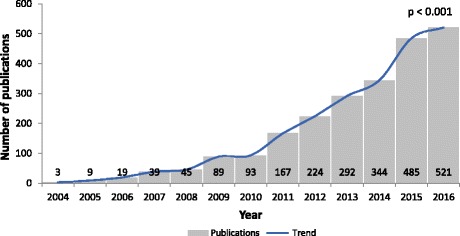
Annual scientific documents of biosimilar research, Scopus 2004–2016

A total number of 2330 documents indexed on Scopus were retrieved from 2004 to 2016. The majority of papers were articles (1452; 62.32%) followed by reviews (642; 27.55%), editorials (138; 5.92%), and letters (98; 4.21%). In 2004, three publications were identified compared to 521 in 2016 (Fig. 1); this increase in the amount of papers was statistically significant (*p* < 0.001). In general, 83 countries contributed to research articles about biosimilars. Table 1 listed the top ten countries, which represented 84.12% of the total. Seven of the top ten publishing countries are European. United States was the country with the highest production with 685 (29.40%) documents followed by Germany and UK with 293 (12.58%) and 248 (10.64%), respectively. European countries such as Switzerland (11.05), Netherlands (5.85) and UK (3.83) showed the highest per capita ratio. The highest citation/article ratio were for the Netherlands (28.06), Spain (24.23), and France (20.11).

**Table 1:** Top ten countries with more publications on biosimilars, and there average citation rate. Scopus 2004–2016

Rank	Country	Number of documents	% of articles	Number of articles per million inhabitants	Citations (up 2016)	Citations/Article
1	United States	685	29.40	2.10	6768	9.88
2	Germany	293	12.58	3.64	4373	14.92
3	United Kingdom	248	10.64	3.83	3824	15.42
4	Italy	149	6.39	2.40	2097	14.07
5	France	132	5.67	2.10	2654	20.11
6	Netherlands	100	4.29	5.85	2806	28.06
7	Spain	91	3.91	1.86	2205	24.23
8	Switzerland	91	3.91	11.05	1783	19.59
9	India	86	3.69	0.07	338	3.93
10	Canada	85	3.65	2.39	794	9.34

The analysis of the subject area, Table 2 shows that most documents were included in the broad category “Medicine” with 1453, followed by “Pharmacology, toxicology and pharmaceutics” with 946 and “Biochemistry, Genetics and Molecular biology” with 607.

**Table 2:** Subject areas for documents published on biosimilars, Scopus, 2004–2016

Rank	Subject area	Documents published	% of documents
1	Medicine	1453	37.92
2	Pharmacology, Toxicology and Pharmaceutics	946	24.69
3	Biochemistry, Genetics and Molecular Biology	607	15.84
4	Immunology and Microbiology	219	5.72
5	Health Professions	165	4.31
6	Chemistry	137	3.58
7	Chemical Engineering	136	3.55
8	Business, Management and Accounting	68	1.77
9	Engineering	62	1.62
10	Mathematics	39	1.02

**Table 3:** Top ten journals publishing on biosimilars (*N* = 2330). Scopus 2004–2016

Rank	Source title	Publication type	Documents published	% of articles	CiteScore 2016	SJR 2016	SNIP 2016
1	Gabi Journal	Journal	73	3.13	0.28	0.23	0.08
2	Biopharm International	Trade Publication	41	1.76	0.17	0.16	0.08
3	Mabs	Journal	41	1.76	4.66	1.62	1.25
4	Biodrugs	Journal	40	1.72	2.98	1.01	1.13
5	Bioprocess International	Trade Publication	39	1.67	0.26	0.23	0.25
6	Pharmaceutical Technology	Journal	39	1.67	0.09	0.14	0.16
7	Biologicals	Journal	34	1.46	1.65	0.62	0.74
8	Contract Pharma	Trade Publication	30	1.29	0.02	0.12	0
9	Journal of Generic Medicines	Journal	28	1.20	0.12	0.14	0.39
10	Expert Opinion on Biological Therapy	Journal	27	1.16	3.14	1.14	0.75

The total of documents retrieved were published in 803 journals, but top ten journals account for 18.82% of the total (Table 3). The top journals included in first place *Gabi Journal* with 73 (3.13%) documents, followed by *Biopharm International* and *Pharmaceutical Technology* and *Mabs* both with 41 (1.76%). Three out of ten titles were Trade publications. *Mabs* journal had the greatest SJR 2016 (1.62).

Table 4 presents a ranking of the Top ten institutions that published in biosimilars. Amgen Incorporated from the USA was the most prolific institution with 51 documents followed by Pfizer Inc. with 48. Seven of this top 10 list are European institutions, the others from USA. Four institutions were pharmaceutical companies. Based on the citations, academic institutions obtained higher positions compared to pharmaceutical companies being Medizinische Universitat Wien the institution with the highest number of citations and citations per articles ratio.

**Table 4:** Top ten institutions publishing on biosimilars, and their citations. Scopus 2004–2016

Rank	Institution Name	Country	Number of papers	Citations	Citations/Article
1	Amgen Incorporated	United States	51	394	7.73
2	Pfizer Inc.	United States	48	201	4.19
3	Utrecht University	Netherlands	44	898	20.41
4	Duke University	United States	37	763	20.62
5	Sandoz International GmbH	Germany	33	460	13.94
6	Medizinische Universitat Wien	Austria	23	1538	66.87
7	KU Leuven	Belgium	22	227	10.32
8	Erasmus University Medical Center	Netherlands	20	929	46.45
9	Universita degli Studi di Milano	Italy	20	152	7.6
10	Novartis International AG	Switzerland	19	174	9.

**Fig. 2:**
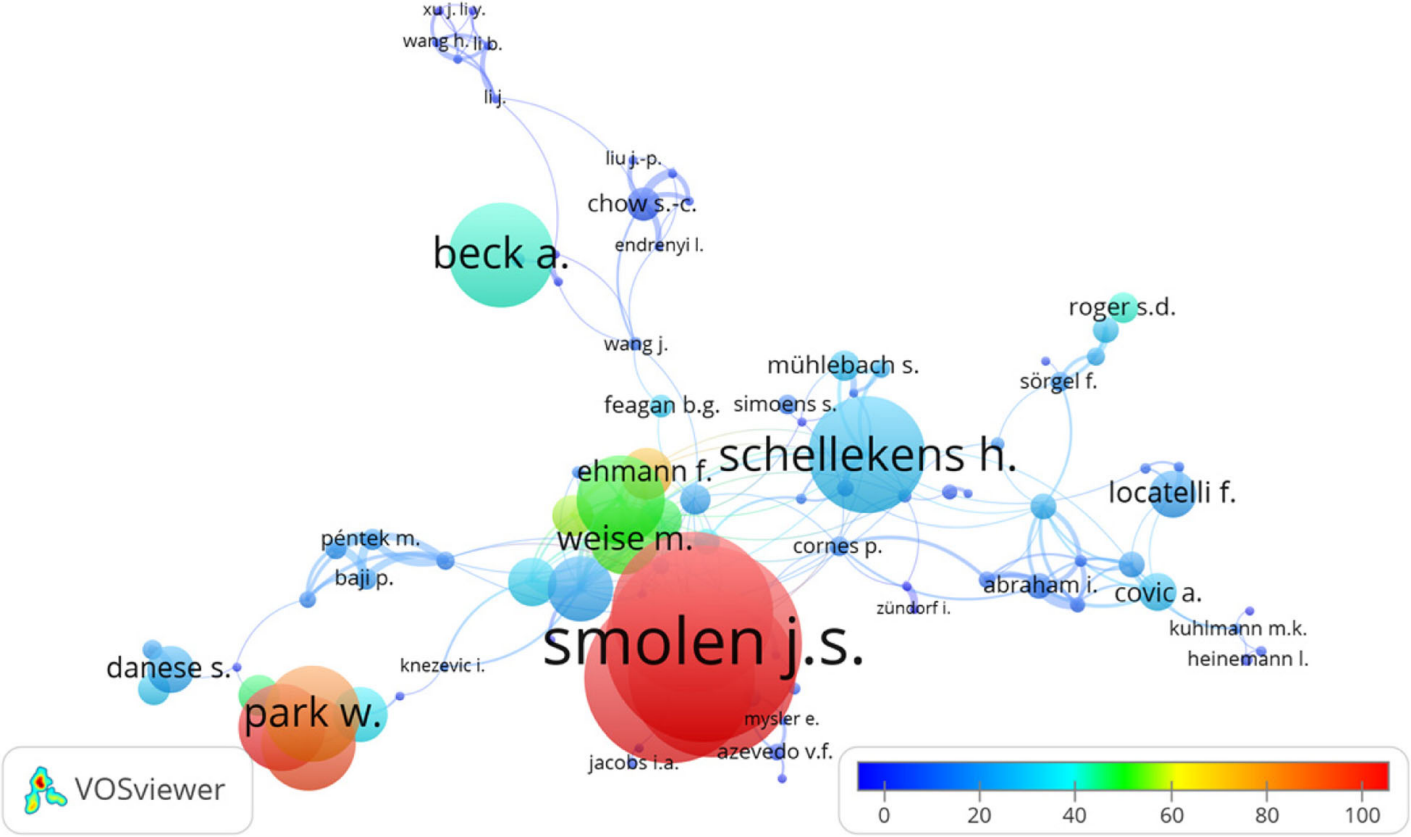
Author co-authorship analysis with VOSviewer for biosimilars publications. Minimum of five documents per author, 143 authors were included. Weighted by citations and scores by average of citations

There were 6344 authors in the documents on biosimilars. The mean of articles per author was 0.4 and there were 420 documents with only one author. A co-authorship analysis that included authors with at least five publications is shown in Fig. 2. There were 143 authors; each circle represents one author, the closer the circles the closer the collaboration.

We show the top 10 cited articles in biosimilars in Table 5. The article “EULAR recommendations for the management of rheumatoid arthritis with synthetic and biological disease-modifying antirheumatic drugs: 2013 update” published in 2014 received the highest citation (962) within the retrieved documents. The top 10 include five articles and five reviews. All of the top ten cited articles were published in scientific journals. In general, the mean of citations per article was 9.6.

**Table 5:** Top 10 cited documents of biosimilars research. Scopus 2004–2016

Rank	Title	Year	Journal name	Cited by	Type of document
1	EULAR recommendations for the management of rheumatoid arthritis with synthetic and biological disease-modifying antirheumatic drugs: 2013 update	2014	Annals of the Rheumatic Diseases	962	Article
2	2010 update of EORTC guidelines for the use of granulocyte-colony stimulating factor to reduce the incidence of chemotherapy-induced febrile neutropenia in adult patients with lymphoproliferative disorders and solid tumours	2011	European Journal of Cancer	498	Article
3	Biopharmaceutical benchmarks 2014	2014	Nature Biotechnology	275	Article
4	A randomised, double-blind, parallel-group study to demonstrate equivalence in efficacy and safety of CT-P13 compared with innovator infliximab when coadministered with methotrexate in patients with active rheumatoid arthritis: The PLANETRA study	2013	Annals of the Rheumatic Diseases	269	Article
5	A randomised, double-blind, multicentre, parallel-group, prospective study comparing the pharmacokinetics, safety, and efficacy of CT-P13 and innovator infliximab in patients with ankylosing spondylitis: The PLANETAS study	2013	Annals of the Rheumatic Diseases	250	Article
6	Analytical tools for characterizing biopharmaceuticals and the implications for biosimilars	2012	Nature Reviews Drug Discovery	215	Review
7	PEG-modified biopharmaceuticals	2009	Expert Opinion on Drug Delivery	201	Review
8	The challenge of biosimilars	2008	Annals of Oncology	178	Review
9	Sublingual immunotherapy: World Allergy Organization position paper 2013 update	2014	World Allergy Organization Journal	172	Review
10	Biosimilars: What clinicians should know	2012	Blood	158	Review

**Fig. 3:**
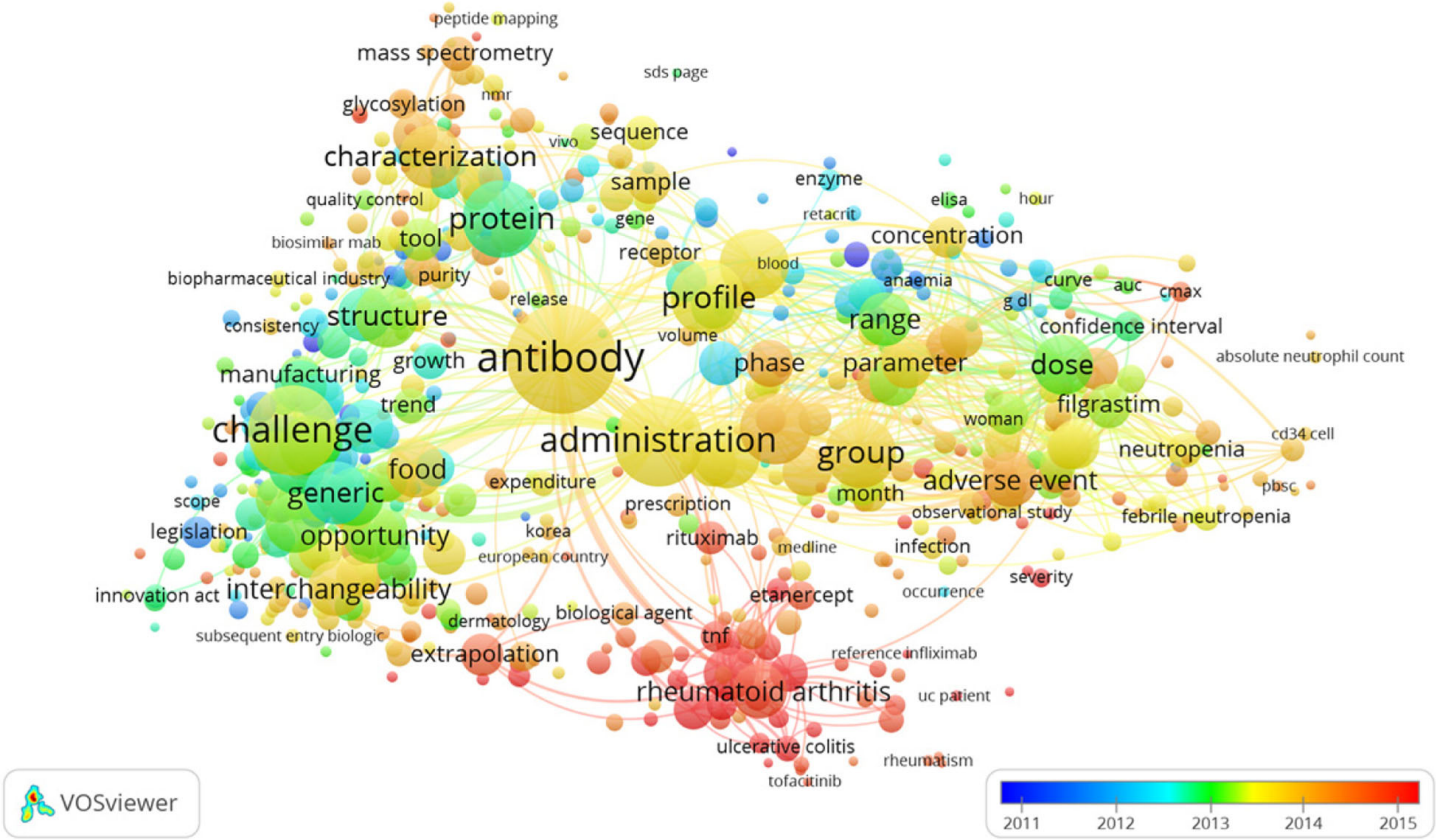
Terms co-occurrence analysis of tittles and abstracts (overlay visualization) and their temporal evolution with VOSviewer for biosimilars publications. Binary counting method, choose threshold (10 terms). Term “conclusion” was excluded

In the analysis of terms co-occurrence (Fig. 3), we used words in the titles and abstracts related to specific diseases and drugs. Rheumatoid arthritis, ulcerative colitis, rheumatism, infliximab, etanercept, and tofacitinib were found in recent years, compared with terms such as legislation, structure, protein, dose and generic in the early years.

## Discussion

Biosimilars’ cost-saving potential is their best attribute, making them an attractive option in the close future [19]. Also, the mandatory research that they have to do prior of their approval, and their large utility in diverse diseases, such as cancer, hemophilia, autoimmune diseases, and rare genetic conditions, make biosimilars an interesting field to make research. This explains the growing interest by many groups, including patients, health insurers, and providers, as well as the pharmaceutical industry. This interest is evidenced in the exponential increased of documents published on biosimilars between 2004 and 2016. High-income countries seem to dominate research in the field of biosimilars where the United States and Western European countries contributed to most of the world’s research on biosimilars, and receive the most of the citations.

Authors from the United States published the highest number of scientific publications and received more citation compared to other countries. This result is not surprising, since the US leads the rankings in worldwide research, including medicine [20–22]. Majority of top-ranking countries were European. Although Switzerland, Netherlands, and United Kingdom do not have as many papers but go to the top of the list when we adjust by population. On the other hand, Netherlands, Spain, and France have the greatest citation per article. Those situations are similar in other biomedical fields: Rheumatology [20], arthroscopy [23], foot and ankle [24], and probiotics in pediatrics [25], but it differs from endocrinology and metabolism field [26] and research on tramadol [27]. It would perhaps be better to adjust by the number of researchers, instead of inhabitants, but this information is not easily accessible [26].

“Gabi Journal” and “Mabs” were the most productive peer-review journals in the topic of biosimilars. Besides, “Biopharm International”, a trade publication journal, occupied the second most productive journal on biosimilars. It is remarkable that three trade publication journals, perhaps showing the industry’s interest in promoting their products, were in the top 10 most productive journals, but they do not receive that many citations. This may reflect the fact that they might be considered less trustful, or that they are not necessarily targeted at researchers [28]. Both “Mabs” and “Expert Opinion on Biological Therapy” are rated much higher in CiteScore 2017, SJR 2016, and SNIP 2016, despite having less publications. These suggest that at least in the field of biosimilars quantity is not necessarily correlated with quality. This situation differs from other biomedical fields like spine surgery [29].

Only three of the top 10 institutions were from America, the other seven were from Europe. This reflects the fact that biosimilars are a subject of interest in many different countries. Additionally, institutions with most citations and mean citation rate were from universities, which might indicate its better quality. Also, there is less risk that universities have conflicts of interest in developing research papers in biosimilars, so their results might be more trustful.

The top five most cited papers were original articles. The top two were guidelines for management of clinical conditions (rheumatoid arthritis and febrile neutropenia). This could be explained because three biosimilars are available to treat rheumatoid arthritis and others are in the way to approval [30], and the recent approval process of granulocyte-colony stimulating factor biosimilars, a useful drug to treat febrile neutropenia [31]. Moreover, clinical practice guidelines from any field generally have a lot of cites. The fourth and fifth most cited articles were randomized controlled clinical trials of the infliximab biosimilar CT-P13. This is the first biosimilar monoclonal antibody approved by the European Medicines Agency [32]. Overall, those two clinical trials help to understand and accept their interchangeability with infliximab as a feasible and safe strategy to be applied in real-life clinical practice [33]. On the other side, there were five reviews in the top 10. Mostly, they try to better explain biosimilars to health professionals, especially clinician [34], that could have some reluctance about the use of biosimilars [35]. These reviews focus on improving the uptake of biosimilars, educating the physicians, and motivating to adopt them in routine clinical practice [36].

Words included in the title and abstracts of biosimilars research papers and their main year when they were published were: Legislation (2011), enzyme (2012), structure, protein, dose, antibody, interchangeability (2013), glycosylation, receptor, phase, adverse event (2014), and rheumatoid arthritis, ulcerative colitis, extrapolation, rituximab, etanercept (2015). This suggests that the emphasis of biosimilars research responds to a subject that is very new to researchers and should follow the law of the development of a new discipline. This find is similar to a report in a previous bibliometric analysis on exomes [37]. In the field of biosimilars, there are requirements for their approval, including to demonstrate similarity, safety, and effectiveness [38]. These terms were more frequently found in the early.

The present research shows an overall view about biosimilars research and their distribution mostly in high-income countries where they have policies to their approval. Although markets like BRICS (Brazil, Russia, India, China, and South Africa), MIST (Mexico, Indonesia, South Korea, and Turkey) [39], and Latin America [40] provides an emerging future to biosimilars, they do not represent an important influence in biosimilars research. Also, the present study like previous bibliometric analyses has some limitations. First, our study did not include articles published in non-Scopus databases. However, Scopus is a reliable and significant source for bibliometric studies in general. Second, it is difficult to distinguish articles that focus on biosimilars from those that only mention the term or tangentially address it. However, using correct keywords and given its original subject, this study still provides a comprehensive picture of biosimilar research productivity, which could be used to track overall trends and identify topics of interest.

## Conclusions

In summary, the results of the present study showed that documents published in journals about biosimilars are increasing. The majority of publications came from high-income countries, being the US the most productive country in biosimilars followed by European countries. To the best of our knowledge, this is the first study conducted in the analysis of research production and citations in biosimilars. The trends in terminology use are according to state of the art in the topic that comes from issues of safety and efficacy to the study of new biosimilar products in specific diseases.

## Additional file

Additional file 1: Dataset 1: Data obtained from Scopus, a .CSV that contain list of studies included. (CSV 6323 kb)

## Abbreviations

MeSH: Medical subject heading
SNIP: SCImago journal rank
SRJ: Source normalised impact per paper
UK: United Kingdom
USA: United States of America
